# Formation of different flavor characteristics of raw- and boiled-dried oysters

**DOI:** 10.1016/j.fochx.2025.102854

**Published:** 2025-07-30

**Authors:** Duanquan Lin, Yu-Lei Chen, Wei-Sen Huo, Jing-Yi Wang, Ling-Jing Zhang, Jia-Yin Huang, Le-Chang Sun

**Affiliations:** College of Ocean Food and Biological Engineering, Jimei University, Xiamen, China

**Keywords:** Dried oyster, Flavor formation, Protein degradation, Glutamic acid release

## Abstract

The release of flavor compounds in dried oysters, particularly umami amino acids, is highly dependent on the proteolytic degradation efficiency of specific targeted proteins, which is critically influenced by processing techniques. However, systematic studies on this mechanism remain scarce. This study elucidated how processing techniques govern flavor formation in dried oysters. Mild drying 50 °C in raw oysters triggered intense protein degradation, increasing glutamic acid and maximizing umami intensity, but accumulated bitter compounds. Conversely, 90 °C drying suppressed bitterness yet reduced umami and caused extreme yellowing. Mass spectrometry revealed that hydrophilic domains in ∼35 kDa oyster proteins (mainly glutamic acid-rich proteins, such as *β*-tubulin chain and actin-like proteins) were enzymatically targeted, releasing free glutamic acid as the core umami contributor. This work demonstrated the synergistic effects of pre-cooking and temperature on flavor, and findings provide a scientific basis for optimizing dried oyster processing.

## Highlights

• Pre-treatment and hot-drying temperature shaped flavor in dried oysters.
• Pre-boiling of oysters eliminated sourness and created balanced umami-bitterness.
• Gentle drying at 50 °C in raw oysters maximized umami intensity.
• Degrading glutamate-rich proteins during drying was critical for umami formation.
• Drying oysters at 90 °C minimized bitterness but sacrificed umami.

## Graphical abstract

**Figure:**
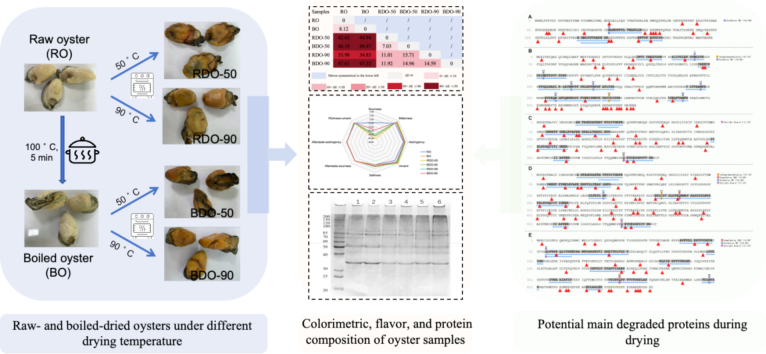

## 1 Introduction

Oysters are highly favored by consumers worldwide, due to their rich nutritional profile and palatable taste (Iitembu et al., 2023). In particular, the Portuguese oyster (*Magallana angulata*), widely cultivated along China's coast, represents a key commercial species prized for its distinct umami flavor and texture. However, their high moisture content and abundant nutrients render them highly susceptible to spoilage during production, transportation, and storage under external influences such as microbial activity and temperature fluctuations, ultimately leading to the loss of edibility (Watakabe et al., 2025). Consequently, in Asian countries like China, drying processes are employed to extend the shelf-life of oysters (Zhao et al., 2023). Dried oyster products have gained widespread popularity in Asian markets, owing to their distinctive flavor and portability (Li et al., 2025).

Two primary methods exist for producing dried oysters: The more common approach involves boiling fresh oyster meat followed by sun-drying or hot-air drying, yielding boiled-dried oysters (BDO) (also named as cooked-dried oysters) (Wang et al., 2023); Alternatively, raw-dried oysters (RDO) are produced by directly drying unboiled fresh oyster meats (Neri et al., 2021). Conventional drying techniques for BDO often trigger undesirable color deterioration (e.g., browning and darkening) and flavor imbalance (e.g., insufficient umami), severely constraining industrial upgrading (Hu et al., 2024). This quality degradation originates from complex physicochemical changes during drying, mainly including the interactions among protein denaturation, enzymatic reactions, and non-enzymatic browning (Bonazzi & Dumoulin, 2011; Hu et al., 2024). In addition, it has been found that drying fresh oysters using hybrid pump dryer at 55 °C/10 h + 45 °C/14 h or 65 °C/7 h + 45 °C/17 h showed exhibited lesser browning activity, better surface color, and higher antioxidant than those using hot air dryer at 70 °C/24 h, indicating that different drying processes show various effects on the final quality of RDO (Neri et al., 2021). Studies also indicated that oysters harbor diverse endogenous enzymes, such as serine proteases and cysteine proteases, which critically influence protein degradation, autolytic softening, and quality deterioration during refrigeration (Su et al., 2024). It is therefore hypothesized that these endogenous enzymes also play a pivotal role in developing the umami-sweet taste profile of RDO. Throughout the drying process, sustained degradation of oyster proteins by endogenous enzymes generates abundant peptides and amino acids, thereby enhancing the palatability of RDO (Neri et al., 2021; Su et al., 2024). Although prior research has examined the impact of isolated factors (e.g., temperature and humidity) on texture or volatile compounds of dried oysters (Cheng et al., 2017; Liu et al., 2024; Neri et al., 2021), the core scientific question remains unresolved: How drying temperature synergistically drives the color evolution and flavor formation of dried oysters by modulating the endogenous enzyme activity and protein stability.

A critical shortage in current research is the lack of systematic investigation into the interconnected mechanisms governing multidimensional quality indicators of dried oysters. On one hand, color changes involve the coupled effects of Maillard reaction kinetics, carotenoid transformation, and microstructural collapse (Hu et al., 2024). On the other hand, the release of flavor compounds, particularly umami amino acids, is highly dependent on the proteolytic degradation efficiency of specific targeted proteins (Su et al., 2024). Notably, pre-cooking (normally pre-boiling), as a common pretreatment, may profoundly alter the final product's quality profile by modifying the activity of enzyme systems and the availability of substrates. Consequently, elucidating the synergistic effects of pre-boiling and drying temperature is essential for the precise regulation of dried oyster quality.

Given that conventional drying techniques cause severe browning and flavor imbalance in BDO, and the core scientific question (i.e., how drying temperature synergistically regulates endogenous enzyme activity and protein stability to drive flavor formation) remains unresolved, this study's systematic investigation into the interconnected mechanisms governing multidimensional quality indicators is urgently necessary to prevent industrial upgrading constraints. At the macro level, chromaticity and electronic tongue techniques quantified the evolution of color and taste parameters. At the molecular level, amino acid quantification coupled with proteomics tracked the release of key flavor precursors (e.g., glutamic acid) and revealed their correlation with protein degradation. The experimental design contrasted raw and boiled oysters followed by drying at 50 °C or 90 °C to decipher the differential regulatory mechanisms of temperature on the protein stability and the release of flavor compounds. The theoretical significance lies in establishing, for the first time, a ‘temperature-enzyme activity-protein degradation-flavor’ cascade regulation model, which may fill critical theoretical gaps in understanding the mechanisms of the release of flavor precursors e.g., glutamic acid, providing vital scientific support for precise quality control of products.

## 2 Materials and methods

### 2.1 Materials

Fresh shucked oysters (*Magallana angulata*) were purchased from PUPU supermarket (Xiamen, Fujian, China) and immediately transported to the laboratory under cold-chain conditions. Analytical-grade reagents, including ammonium bicarbonate, dithiothreitol (DTT), iodoacetamide (IAA), acetonitrile (ACN), formic acid (FA), NaOH, HCl and others, were procured from Sigma-Aldrich (St.Louis, MO, USA).

### 2.2 Drying kinetics study

Fresh oysters of comparable size with length of 5.23 ± 0.44 cm, width of 3.08 ± 0.25 cm, and weight of 11.44 ± 1.26 g were selected. Half were boiled in water at 100 °C for 5 min, quenched in ice-water, drained, and surface-dried with filter paper (designated BO). The remaining half were surface-dried without pretreatment (designated RO). Both BO and RO groups were further subdivided for mild drying at 50 °C (designated BDO-50 and RDO-50, respectively) or high-temperature drying at 90 °C (designated BDO-90 and RDO-90, respectively). Samples were evenly distributed on drying trays and placed in a preheated drying oven with air flow velocity of 2.0 m/s. Moisture content (MC) was monitored gravimetrically every 2 h for 18 h at 50 °C and every 30 min for 4.5 h at 90 °C. Moisture content was calculated as following:

MC = (W_0_ − W_c_) / W_0_ × 100 %,

where W_0_ = initial weight, W_c_ = constant weight after drying at 105 °C (Cheng et al., 2017). Drying curves were plotted moisture content vs. time and fitted to kinetic models (Fig. S1).

### 2.3 Preparation of dried oysters

After the pretreatment as described in Section 2.2, oyster samples were then dried at 50 °C or 90 °C to a uniform moisture content of 40 %, based on the kinetic equations as shown in Fig. S1. Drying durations of groups RDO-50, BDO-50, RDO-90, and BDO-90 were 16.8 h, 15.4 h, 3.9 h, and 3.8 h, respectively. Dried samples were finally cooled, sealed in bags, and stored at 4 °C for further investigation.

### 2.4 Colorimetric analysis

Dried oysters were photographed on white A4 papers using an iPhone 12 (Apple Inc., USA). Color parameters including lightness (L*), red-green (a*), and yellow-blue (b*) were measured with a chroma meter (NR60CP, 3NH Technology Inc., Shenzhen, China). Total color difference (Δ*E*) between groups was calculated as:

$$∆E=\sqrt{{\left({L_{1}}^{*}-{L_{2}}^{*}\right)}^{2}+{\left({a_{1}}^{*}-{a_{2}}^{*}\right)}^{2}+{\left({b_{1}}^{*}-{b_{2}}^{*}\right)}^{2}}.$$

### 2.5 Scanning electron microscopy (SEM)

Samples were freeze-dried, and muscle tissues from the mid-region were mounted on aluminum stubs with carbon tape. Gold sputtering (15 mA, 60 s) was performed before observation under a Phenom Pro SEM at 5 kV (ThermoFisher Scientific, USA).

### 2.6 Electronic tongue analysis

Samples were homogenized with deionized water 1:5 (*w*/*v*), followed by centrifuging then and filtering with 0.45 μm filter membranes. The samples were analyzed using a SA402B electronic tongue (Insent, Japan). Sensors were activated in cleaning solutions for 90 s (anode cleaning solution: 100 mM hydrochloric acid +30 % *v*/*v* ethanol; cathode cleaning solution: 10 mM potassium hydroxide +100 mM potassium chloride +30 % *v*/*v* ethanol), and then rinsed twice with a reference solution (30 mM KCl + 0.3 mM tartaricacid) for 120 s each, and finally zeroed for 30 s. Taste attributes of samples (including bitterness, astringency, sourness, saltiness, and umami) were measured for 30 s, followed by aftertaste analysis for 30s after a 3-s rinse.

### 2.7 Amino acid profiling by LC-MS/MS

Samples (100 mg) were mixed with 10 μL of 10 % sulfosalicylic acid and diluted to 1 mL with 0.1 % formic acid. The mixtures were then bead-homogenized for 3 min and sonicated for 15 min. Following centrifugation at 12,000 rpm and 4 °C for 15 min, the supernatants were filtered through a 0.22 μm membrane filter for analysis by liquid chromatography-tandem mass spectrometry (LC − MS/MS; TSQAltis, ThermoScientific, USA).

LC-MS/MS analysis was performed using a Kinetex PFP column (4.6 × 250 mm, 5 μm) maintained at 40 °C. The mobile phases A and B consisted of 10 mM ammonium formate + 0.1 % formic acid in water, and methanol: water 9: 1, *v*/v + 10 mM ammonium formate + 0.1 % formic acid, respectively. Quantitation employed multiple reaction monitoring (MRM) in positive-ion mode with external calibration. Compound identification was achieved by matching retention times and MRM fragment ions against reference standards (Fig. S2).

### 2.8 Analysis on the endogenous enzyme activities of oysters

An appropriate amount of raw or boiled oyster abdominal muscle was taken, followed by adding four times its mass-volume of either Buffer A (20 mmol/L Tris−HCl, pH 8.0) for serinep roteasea ctivity assay or Buffer B (100 mmol/L sodium acetate buffer, pH 5.5) for cathepsin L activity assay. All samples were homogenized for 15 min, followed by centrifugation at 12,000 ×*g* for 20 min at 4 °C. The supernatant was then collected as the crude oyster enzyme extract.

For enzyme activity determination, serine protease and cathepsin L activities were assayed using Boc-Phe-Ser-Arg-MCA and *Z*-Phe-Arg-MCA as substrates, respectively. The crude enzyme extract (50 μL) was firstly added to 900 μL of the corresponding Buffer A or Buffer B, followed by adding 50 μL of 10 μmol/L substrate solution. After thorough vortex mixing, the mixture was incubated at 37 °C for 10 min, then the reaction was immediately terminated by adding 1.5 mL of stop solution (methanol: n − butanol: distilledwater = 35: 30: 35, *v*/v/v). The fluorescence intensity of released 7-amino-4-methylcoumarin (AMC) was then measured using a fluorescence spectrophotometer at 380 nm excitation and 450 nm emission wavelengths. Enzyme activity (U) is defined as the amount of enzyme required to release 1 nmol of AMC per minute. A blank control was prepared identically but with buffer replacing the enzyme solution.

### 2.9 Protein degradation analysis by sodium dodecyl sulfate-polyacrylamide gel electrophoresis (SDS − PAGE)

The sample (1.0 g) was mixed with 10 volumes of deionized water and homogenized by vortexing. Subsequently, 200 μL of the resulting homogenate was combined with 4 volumes of solubilization buffer (20 mM Tris-HCl (pH 8.0), 8 M urea, 1 % SDS and 2 % *β*-mercaptoethanol) and then shaken until complete dissolution. Protein concentration was adjusted to 2 mg/mL spectrophotometrically, followed by denaturation at 95 °C for 5 min. For electrophoresis, 10 μL of the processed sample was loaded onto a 12 % separating gel prepared. Electrophoresis was performed at 80 V through the stacking gel and 120 V through the separating gel. The process was terminated when the bromophenol blue dye front reached the gel bottom, followed by Coomassie Brilliant Blue staining for 30 min. Destaining was conducted using a solution of 30 % methanol and 10 % acetic acid until band edges became colorless. Finally, gel images were acquired using a G:BOX gel documentation system (Syngene, UK) for protein molecular weight analysis.

### 2.10 Identification of degraded proteins by LC-MS/MS

Following washing with ddH_2_O three times, targeted protein bands were destained with 150 μL destaining solution and rinsed four times with water. Sequential washes were performed with 300 μL of 25 mM ammonium bicarbonate, 50 % acetonitrile ACN, and 100 % ACN until gel pieces attained an opaque appearance. Reduction was conducted by adding 50 μL of 10 mM DTT solution and incubating at 56 °C for 30 min. After cooling to room temperature, alkylation proceeded in darkness with an equal volume of 50 mM IAA solution for 15 min. The gradient washing cycle was performed with 300 μL of 25 mM ammonium bicarbonate, 50 % acetonitrile ACN, and 100 % ACN with dehydration until opacity was achieved. For in-gel digestion, gel pieces were rehydrated on ice with 15–20 μL of 0.01 μg/μL trypsin until transparent, then overlaid with 30–40 μL of 50 mM NH_4_HCO_3_ containing 10 % ACN. Enzymatic digestion proceeded at 37 °C for 24 h. The resulting supernatant was transferred to a fresh microcentrifuge tube, while residual gel pieces underwent extraction with 100 μL extraction buffer (67 % acetonitrile containing 2 % FA) at 37 °C for 30 min, followed by 15-min ultrasonication and centrifugation. Combined supernatants were concentrated via centrifugal evaporation for subsequent mass spectrometry analysis.

Digested peptides were reconstituted in Nano-LC mobile phase A (0.1 % FA in water) and loaded onto a nanoViper C18 trap column (3 μm, 100 Å). Chromatographic separation was performed using an Easy-nLC 1200 system (ThermoFisher Scientific, USA) equipped with an Acclaim PepMap RSLC C18 analytical column (75 μm × 25 cm, 2 μm, 100 Å), applying a 60-min linear gradient from 5 % to 40 % of mobile phase B (80 % acetonitrile containing 0.1 % FA). Mass spectrometry analysis employed an Orbitrap Eclipse system (ThermoFisher Scientific, USA) with NanoFlex ion source operated at 2 kV spray voltage and 320 °C of an ion transfer tube.

The raw mass spectrometry data were processed using PEAKS Online 11 software, with database searches performed against the Magallana angulata protein database downloaded from NCBI. Search parameters were specified as follows: tryptic digestion allowing a maximum of two missed cleavages; precursor mass tolerance of 10 ppm and fragment mass tolerance of 0.05 Da; fixed modifications included carbamidomethylation of cysteine residues, while variable modifications encompassed N-terminal acetylation, deamidation of asparagine/glutamine residues, oxidation of methionine, and pyro-glutamic acid formation from glutamate/glutamine.

### 2.11 Statistical analysis

Statistical analyses in this study were conducted using IBM SPSS Statistics 27 software (IBM Corp., USA). Significant differences among datasets were evaluated through Tukey's HSD post-hoc test, with results expressed as mean ± standard deviation (*n* = 3). Statistical significance was defined at *p* < 0.05. All graphical representations of data were generated using Origin 2021 (OriginLab Corp., USA).

## 3 Results and discussion

### 3.1 Morphological characteristics of dried oysters

**Fig. 1:**
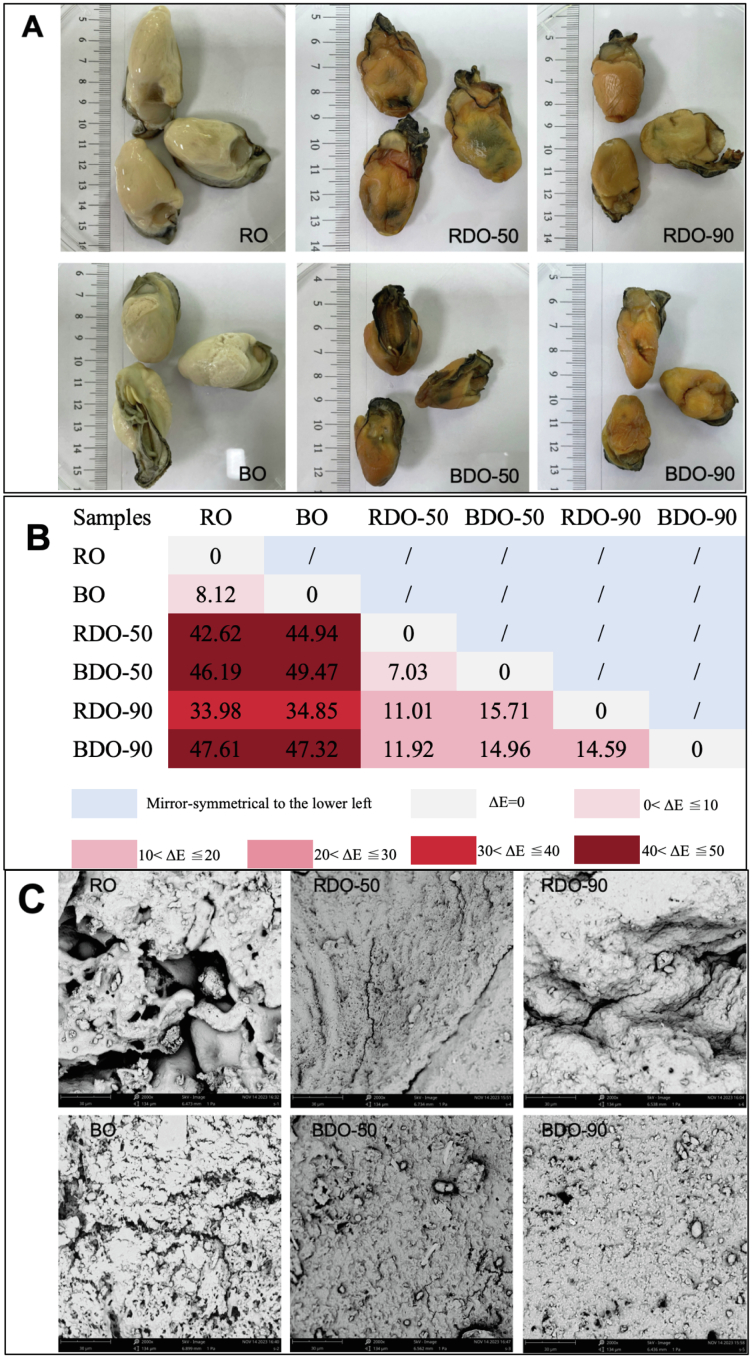
**Morphological characteristics of oysters**: (A) Appearance of oyster samples, including raw oyster (RO), boiled oyster (BO), and their dried products under mild drying at 50 °C (designated RDO-50 and BDO-50, respectively) or high-temperature drying at 90 °C (designated RDO-90 and BDO-90, respectively); (B) Total color difference (Δ*E*) between groups; (C) Micro-structrues (SEM) of oyster samples.

Firstly, the apparent differences between raw oysters (RO) and boiled oysters (BO) was compared. The results from Fig. 1A and Table 1 indicate that RO and BO exhibited significant differences in color parameters. The L* value of oysters after boiling increased from 77.05 to 82.13, indicating a noticeable whitening and brightening of the tissues; The a* value slightly decreased from 1.76 to 1.54, but the difference was not significant, suggesting no substantial change in red-green tendency; While the b* value dramatically increased from 14.01 to 20.43, reflecting a significant yellow bias in boiled oysters.

**Table 1:** Colorimetric analysis of oyster samples (color parameters including lightness (L*), red-green (a*), and yellow-blue (b*)).

**Samples**	L*	a*	b*
**RO**	77.05 ± 3.56^a^	1.76 ± 0.79^c^	14.01 ± 1.04^c^
**BO**	82.13 ± 5.41^a^	1.54 ± 0.68^c^	20.43 ± 2.54^c^
**RDO-50**	42.13 ± 1.41^bc^	12.48 ± 1.21^a^	35.96 ± 2.70^b^
**BDO-50**	35.70 ± 3.70^c^	9.68 ± 2.72^a^	35.43 ± 2.85^b^
**RDO-90**	50.96 ± 3.13^b^	5.93 ± 0.54^b^	35.38 ± 2.23^b^
**BDO-90**	44.44 ± 0.96^bc^	10.73 ± 2.41^a^	47.52 ± 6.02^a^

Values are given as mean ± standard deviation (SD, *n* = 3), and different letters in the same column indicate significantly different (*p* < 0.05).

The increase in L* value of oysters after boiling is primarily attributed to physicochemical changes during heating, because boiling water causes rapid denaturation and coagulation of oyster proteins (e.g., myosin), leading to enhanced light scattering (You et al., 2025). Additionally, tissue contraction promotes the loss of free water, increasing the concentration of solid substances and thereby enhancing light reflectance, a mechanism analogous to the whitening of egg whites during boiling (Eregama et al., 2024; Xue et al., 2021). The increase in b* value of oysters after boiling may be dominated by two biochemical reactions: Firstly, the Maillard reaction is activated at high temperatures, where free amino acids (e.g., arginine) in oysters combine with reducing sugars (e.g., glucose) to form melanoidin pigments (Jiang et al., 2021); Secondly, heating disrupts cell membrane structures, releasing bound carotenoids, such as astaxanthin and lutein (Zhang et al., 2019).

After hot-air drying, RO underwent drastic color changes, and the drying temperature significantly influenced the direction of color evolution (Fig. 1A and Table 1). RO samples dried at 50 °C (RDO-50) exhibited a dark reddish-yellow hue, characterized by significantly decreased L* value and significantly increased a* and b* values, showing a color difference Δ*E* as high as 42.62 (Fig. 1B), compared to RO. In contrast, samples dried at 90 °C (RDO-90) displayed a bright yellowish-brown appearance. Although the changes of all three color parameters followed the same trend as those of RDO-50 with a similar magnitude of change in b* value, RDO-90 showed smaller reductions in L* value and smaller increases in a* value, with a Δ*E* of 33.98 (Fig. 1B). This indicates that 50 °C drying intensified darkness and enhances reddening in RO, while 90 °C drying preserved partial brightness and weakens reddening, though both temperatures trigger intense yellowing.

The decrease in L* values of RDO-50 and RDO-90, compared to that of RO, primarily results from moisture removal and microstructural collapse. During drying, the moisture content of oysters dropped from approximately 90 % to 40 %, causing the network to contract and form a dense porous structure, significantly enhancing light absorption efficiency (Fig. 1C). At 50 °C, the slow drying rate created finer micropores, leading to extremely low L* values, whereas rapid dehydration at 90 °C generated larger pores, resulting in relatively higher light reflectance (Fig. 1C). The divergent reddening behavior (i.e., the increase in a* value) may be regulated by non-enzymatic browning kinetics (Hu et al., 2024). The 50 °C condition (i.e., slow drying) allows sufficient reaction between free amino acids (e.g., arginine) and reducing sugars in oysters, continuously generating reddish-brown melanoidin pigments, which causes a sharp increase in a* value for RDO-50. At 90 °C, the short drying time and high initial moisture content briefly inhibits the Maillard reaction, while partial thermal decomposition of pigment precursors reduces the increase in the a* value of RDO-90 (Sharayei et al., 2024; Sherwin & Labuza, 2006). The surge in yellowness (i.e., b* value) of RDO-50 and RDO-90, compared to that of RO, probably stems from both carotenoid concentration and chemical transformation. Moisture loss during drying concentrates natural pigments like astaxanthin and lutein, while thermal oxidation induces isomerization of lutein with change of color from yellow to colorless (Updike & Schwartz, 2003). This effect primarily depends on the total thermal input (i.e., time × temperature). Since the final moisture contents of samples were similar for both 50 °C and 90 °C drying at endpoints, their b* values showed no significant difference.

BO also underwent drastic color changes after hot-air drying, with the drying temperature exhibiting a nonlinear effect on coloration. Samples dried at 50 °C (BDO-50) displayed a dark reddish-yellow hue, characterized by significantly decreased L* value, alongside markedly increased a* and b* values (Table 1). The color difference Δ*E* between BO and BDO-50 reached 49.47 (Fig. 1B). In contrast, samples dried at 90 °C (BDO-90) exhibited a bright orange-yellow appearance. However, compared to BDO-50, the reduction in L* value of BDO-90 was less pronounced, the increase in a* value is similar, while the increase in b* value became exceptionally amplified (Table 1), resulting in a Δ*E* of 47.32 (compared to BO) (Fig. 1B). This indicates that drying at 50 °C produced extremely dark coloration with moderate red-yellow shifts, whereas drying at 90 °C retained slightly higher lightness but triggered intense reddening and extreme yellowing.

The decrease in L* value probably primarily results from the dual dehydration effects of boiling and drying. BO already lost partial moisture during boiling, and hot-air drying further reduced water content (Fig. S1). Slow drying at 50 °C caused continuous contraction of the protein matrix, forming an ultra-dense microporous structure (Fig. 1C). Rapid dehydration at 90 °C generated larger pores, where residual pore water created faint light reflection, leading to a marginally higher L* value (Fig. 1C). Enhanced reddening is driven by the synergistic effect of pre-boiling and drying. The Maillard reaction initiates during boiling with b* increasing from 14.01 to 20.43, and continues to slowly generate reddish-brown melanoidins during 50 °C drying. However, at 90 °C, protein denaturation and cell membrane rupture from pre-boiling release abundant free amino acids and reducing sugars, triggering explosive browning during high-temperature drying and causing a sharp surge in a* value (Ajandouz et al., 2008). Extreme yellowing is fundamentally attributed to carotenoid oxidation and polymerization (Mogg & Burton, 2021). Astaxanthin and lutein released during BO boiling become concentrated during drying. Moderate oxidation occurs at 50 °C, while the high temperature (i.e., 90 °C) accelerates pigment reactions through multiple mechanisms, including isomerization of lutein, oxidative polymerization of astaxanthin, and byproducts of the Maillard reaction.

### 3.2 Gustatory analysis of dried oysters

Results in Table. 2 indicate significant differences in the taste profiles between RO and BO. RO exhibited a sour-salty-dominated flavor profile, with both sourness and saltiness values exceeding the taste recognition threshold, while bitterness, astringency, and umami fell below the recognition threshold. After boiling for 5 min, BO underwent a fundamental flavor transformation. Its sourness disappeared, saltiness remained stable, and bitterness, astringency, and umami all crossed the recognition threshold, forming a new flavor system characterized by balanced umami-bitterness with mild astringency. The disappearance of sourness probably stems from volatile loss of organic acids and an increase in pH. Small-molecular acids in RO (e.g., succinic acid and pyruvic acid) volatilize extensively during boiling (Liu et al., 2021), while protein denaturation releases alkaline amino acids (e.g., arginine), elevating tissue pH (Table S1). The emergence of bitterness and astringency may be attributed to protein hydrolysis generating hydrophobic bitter peptides during boiling, alongside oxidation and polymerization of polyphenols forming aggregates like ellagic acid, coupled with the release of metal ions (e.g., zinc and iron) (Song et al., 2024). The dramatic increase in umami intensity and richness is primarily driven by nucleotide conversion and synergistic effects. On the one hand, thermal activation of AMP deaminase promotes the conversion of adenosine monophosphate (AMP) to inosine monophosphate (IMP) (Wang et al., 2023). On the other hand, protein hydrolysis liberates glutamic acid (Glu) (Table S1). These compounds synergistically interact with umami receptors. Saltiness stability likely results from the thermal inertness of inorganic salts, because sodium, potassium, and other ions exhibit high water solubility and their leaching rates during boiling reach a balance with water loss, while glycogen colloids in oyster tissue also inhibit ion diffusion, maintaining saltiness consistently above the recognition threshold with only minor fluctuations (Liu et al., 2021).

**Table 2:** **Taste attributes of oyster samples analyzed by electronic tongue.** (Note: The tasteless points for sourness and saltiness are −13 and − 6, respectively, while the tasteless point for all other tastes is 0. Based on these reference thresholds, when a sample's taste value falls below its tasteless point, it indicates the absence of that taste, and conversely, values above the threshold confirm the presence of that taste characteristic.)

Samples	Sourness	Bitterness	Astringency	Umami	Saltiness	Aftertaste-sourness	Aftertaste-astringency	Richness-umami
RO	−9.33 ± 0.87^a^	−0.27 ± 0.22^d^	−0.46 ± 0.33^d^	−1.05 ± 0.07^e^	−5.33 ± 0.04^d^	−0.18 ± 0.28^a^	−0.52 ± 0.37^a^	0.05 ± 0.06^d^
BO	−27.24 ± 0.92^dc^	1.89 ± 0.18^bc^	0.73 ± 0.28^c^	2.26 ± 0.26^cd^	−5.05 ± 0.03^c^	−0.32 ± 0.11^ab^	−0.51 ± 0.15^a^	0.57 ± 0.18^c^
RDO-50	−30.62 ± 3.45^d^	3.35 ± 0.67^a^	1.85 ± 1.04^a^	4.43 ± 0.72^a^	−3.64 ± 0.17^b^	−0.55 ± 0.53^b^	−0.89 ± 0.42^c^	1.39 ± 0.33^a^
BDO-50	−24.64 ± 2.61^bc^	2.3 ± 0.16^ab^	1.89 ± 0.28^a^	3.42 ± 0.38^b^	−2.69 ± 0.02^a^	−0.35 ± 0.28^ab^	−0.83 ± 0.25^bc^	0.51 ± 0.11^cd^
RDO-90	−26.7 ± 4.79^bcd^	1.84 ± 0.38^bc^	1.17 ± 0.15^b^	3.13 ± 0.78^bc^	−3.69 ± 0.11^b^	−0.34 ± 0.51^ab^	−0.51 ± 0.53^a^	0.97 ± 0.31^b^
BDO-90	−19.03 ± 2.22^b^	1.53 ± 0.22^c^	1.07 ± 0.2^b^	1.8 ± 0.29^d^	−3.41 ± 0.02^ab^	−0.29 ± 0.14^a^	−0.67 ± 0.19^ab^	0.43 ± 0.07^cd^

Values are given as mean ± standard deviation (SD, n = 3), and different letters in the same column indicate significantly different (*p* < 0.05).

The taste differences among RO and their hot-air-dried products at 50 °C (RDO-50) and 90 °C (RDO-90) are primarily manifested as follows: Compared to RO, drying at 50 °C maximized umami intensity and richness but accompanied intense bitterness and astringency; whereas compared to RDO-50, drying at 90 °C inhibited bitterness generation but incurred significant loss in umami intensity (Table 2). The peak of umami and richness in RDO-50 probably relies on gentle drying preserving enzymatic systems: AMP deaminase could efficiently convert AMP to IMP at 50 °C, synergizing with Glu liberated through protein hydrolysis to enhance umami intensity (Liu et al., 2021). In contrast, umami attenuation in RDO-90 occurred, because high temperatures partially degrade IMP into tasteless inosine, while Maillard reactions consume some free Glu (Liu et al., 2021). In addition, the heightened bitterness and astringency in RDO-50 may stem from prolonged low-temperature drying, which facilitates continuous protease hydrolysis of proteins into bitter peptides (e.g., leucyl-valine) and maximizes polyphenol oxidase activity, catalyzing catechin oxidation into highly astringent polymers (e.g., ellagic acid). Conversely, reduced bitterness and astringency in RDO-90 may result from rapid thermal enzyme inactivation at 90 °C, where Maillard intermediates (e.g., pyrroles) can also mask binding sites on bitter peptide receptors, diminishing perceived bitterness (Liu et al., 2022). Additionally, both RDO-50 and RDO-90 exhibited complete loss of sourness compared to RO, probably due to shared mechanisms, in which organic acids (e.g., succinic acid and pyruvic acid) volatilize with moisture during drying, while protein denaturation releases alkaline amino acids (e.g., arginine) with elevated tissue pH.

Similarly, BO developed a balanced umami-bitterness flavor base after boiling, and drying at 50 °C further enhanced umami intensity and richness, but accompanied intense bitterness and astringency. Compared to BDO-50, drying at 90 °C inhibited bitterness generation but incurred significant loss in umami intensity (Table 2). In summary, both boiled and raw oysters exhibit identical temperature-dependent taste evolution patterns during drying.

**Fig. 2:**
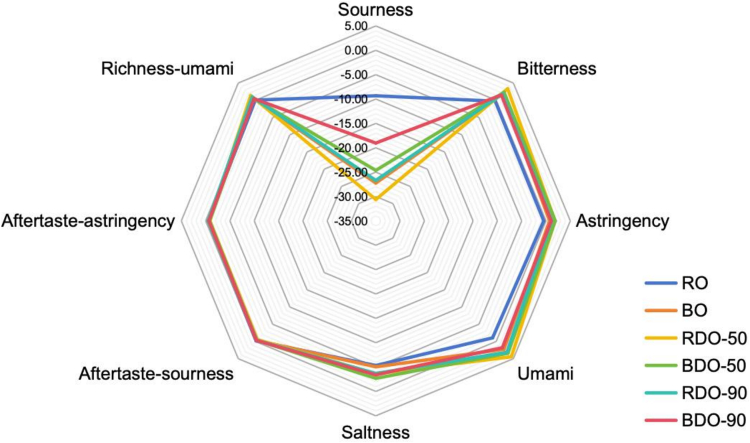
**Gustatory analysis of oysters**: The radar map of flavor profiles (i.e., bitterness, astringency, sourness, saltiness, umami, aftertaste of sourness, aftertaste of astringency, and richness of umani) of oyster samples, including RO, BO, RDO-50, BDO-50, RDO-90, and BDO-90.

In addition, the radar map of flavor profiles in Fig. 2 indicates that among all samples, the largest taste variation occurred in sourness, followed by umami. However, regarding sourness values, only RO with a sourness value of −9.33 slightly exceeded their taste recognition threshold (i.e., −13), while all other samples fell below this threshold, indicating absence of sour taste perception. Therefore, compared to other tastes, umami differences between all samples were actually the most pronounced, reaching maximum values under 50 °C drying conditions (Table 2). Interestingly, the increase level of umami value in BDO-50 (from 2.26 to 3.42) was lower than that in RDO-50 (from −1.05 to 4.43). It was speculated that the primary reason was that although pre-boiling preserves some heat-stable proteases, most proteases become inactivated. In contrast, raw oysters retains abundant proteases that remains active during subsequent gentle drying, effectively degrading proteins to liberate substantial umami amino acids (Luna-González et al., 2004). Therefore, subsequent research would further analyze differences in the amino acid composition and protein degradation profiles among RO/BO and their dried products at 50 °C (i.e., RDO/BDO-50).

### 3.3 Quantitative analysis on amino acids of dried oysters

Regarding total amino acid content, RO contained 4834 μg/g, while after boiling, this value increased by 37 % to 6610 μg/g in BO (Table S1). This increment primarily results from three mechanisms, including concentration effect due to water loss during boiling, protein denaturation and degradation, and partial enzymatic hydrolysis liberating amino acids (Fang et al., 2025). In addition, drying processes significantly impact the liberation of amino acids. When RO was dried at 50 °C (i.e., RDO-50), total amino acids surged to 24244 μg/g (i.e., an approximately 400 % increase, compared to RO). This dramatic rise could be attributed to the full activation of intact protease systems under gentle drying conditions in raw materials. In contrast, pre-boiled oysters dried at 50 °C (i.e., BDO-50) reached 18178 μg/g of total amino acids, a ∼ 170 % increase from BO. This value was significantly lower than that of RDO-50. This discrepancy indicates that pre-boiling at high temperatures has inactivated substantial proteases, severely limiting deep protein hydrolysis efficiency (Benjakul et al., 2023).

**Fig. 3:**
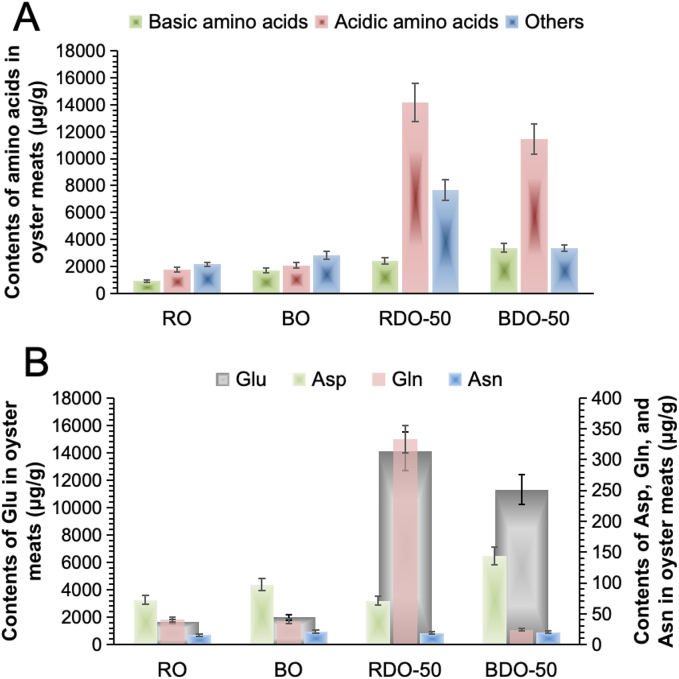
**Contents of amino acids in oyster samples (i.e., RO, BO, RDO-50, and BDO-50)**: (A) Contents of basic, acid and other amino acids (basic amino acids including Lys, Arg, His, Orn and 3MHis with positive charges at physiological pH, acidic amino acids including Asp, Glu and Aad with negative charges at physiological pH, and other amino acids including all other amino acids and analogues listed in the Table 1); (B) Contents of umami amino acids (i.e., Glu, Asp, Gln, and Asn).

Specifically, different types of amino acids exert distinct influences on food flavor profiles (Liu et al., 2023). For instance, acidic amino acids primarily contribute to sourness and umami taste in foods, while neutral polar/non-polar amino acids play significant roles in bitterness. Alkaline amino acids can neutralize acidity but exhibit a positive correlation with bitterness. As shown in Fig. 3A, alkaline amino acids were least abundant in RO (i.e., 924 μg/g), accounting for 19.1 % in total amino acids, yet increased dramatically by 83 % to 1691.3 μg/g after boiling in BO. This aligned with the substantial reduction in sourness observed in BO samples. Alkaline amino acids continued to accumulate during drying, rising to 2428 μg/g in RDO-50 (increase by 163 %, compared to RO), and surging to 3388 μg/g in BDO-50 (increase by 267 %, compared to BO), due to the combined effect of pre-boiling-induced cell membrane disruption and drying concentration. Although excessive alkaline amino acids (particularly arginine) may help neutralize acidity, they can potentially trigger bitter sensations (Melis et al., 2015). Overall, however, alkaline amino acids consistently represented a relatively low proportion of total amino acids across all four samples. Consequently, their concentration does not play a dominant role in shaping the flavor profile of dried oyster products.

The uncharged polar/non-polar and other amino acids in RO had a content of 2156 μg/g with the bitterness value of −0.27 (below the threshold). After boiling (BO), their content increased by 32 % to 2841 μg/g, accompanied by a rise in bitterness value to 1.89, reflecting that boiling promoted the release of hydrophobic amino acids (Fig. 3A). In addition, the drying process exhibited a polarized effect on the release of uncharged amino acids. In RDO-50, uncharged amino acids surged by 255 % to 7643 μg/g (with a bitterness value of 3.35), likely due to the prolonged mild drying facilitating the hydrolysis of hydrophobic amino acids (e.g., Leu) from peptide chains (Table S1; Liu et al., 2023). In contrast, BDO-50 showed only a slight increase of 18 % to 3344 μg/g (with a bitterness value of 2.30), primarily because pre-boiling causes thermal denaturation of proteases, hindering their action on hydrophobic sites.

In contrast, the changes in acidic amino acid content were the most pronounced (Fig. 3A). Acidic amino acids accounted for 36.3 % (1754 μg/g) in RO and increased slightly by 18 % to 2078 μg/g after boiling in BO, directly correlating with the umami value's sharp rise from −1.05 in the raw state to 2.26. The drying process, however, triggered an order-of-magnitude increase. Acidic amino acids in RDO-50 surged by ∼700 % to 14173 μg/g (accounting for 58.5 % of total amino acids), driving its umami value to a peak of 4.43. The key mechanism lies in the continuous hydrolysis of proteins by endogenous proteases (e.g., trypsin) at 50 °C in the raw state, efficiently releasing Glu/Asp (Table S1; Zhang et al., 2024). In contrast, while BDO-50 also saw an increase in acidic amino acids to 11447 μg/g (increase by ∼550 %), its umami value (3.42) remained 23 % lower than that of RDO-50, due to enzyme system damage from pre-boiling. Additionally, some Glu may have been consumed in the Maillard reaction during the pre-boiling stage (Zhang et al., 2021).

**Fig. 4:**
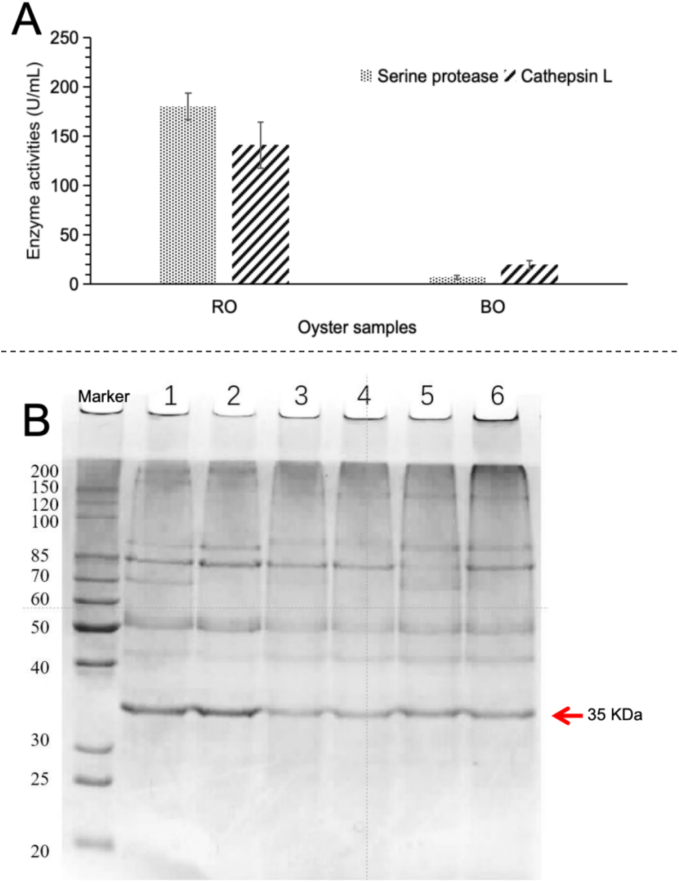
**Endogenous enzyme activities and SDS-PAGE profile of oysters:** (A) The enzyme activities of serine protease and cathepsin L in oyster samples (i.e., RO and BO); (B) SDS-PAGE profile of proteins in oyster samples (lanes 1–6 referring to RO, BO, RDO-50, BDO-50, RDO-90 and BDO-90 samples, respectively).

Moreover, as shown in Fig. 3B, the total umami amino acid content in RO was 1808 μg/g, with Glu being the dominant component (accounting for 92.9 %), reflecting its fundamental role as the primary umami carrier. During boiling (BO), Glu increased by 18 % to 1980 μg/g, attributed to the thermal denaturation-induced release of bound amino acids. In RDO-50, Glu surged to 14100 μg/g (increase by ∼700 %), owing to the prolonged enzymatic hydrolysis at 50 °C, which broke down proteins into glutamine (Gln) and further deamidated it into Glu (Riggs et al., 2019). In contrast, BDO-50 showed a smaller Glu increase to 11300 μg/g (increase by ∼470 %), due to the reduced conversion efficiency from enzyme system damage, as well as partial Glu conversion into Maillard reaction products (e.g., pyrazine volatiles) during pre-boiling. Meanwhile, Gln in RDO-50 sharply increased to 333 μg/g (∼700 % higher than that of raw samples), directly demonstrating the high activity of endogenous glutaminase, which continuously generated Glu precursors. However, in BDO-50, Gln plummeted to 24.1 μg/g (a 40 % decrease), indicating irreversible damage to deamidases caused by pre-boiling. The results in Fig. 4A also proved that the activity of serine protease plummeted from 180.43 U/mL in RO to 7.04 U/mL in BO, representing a 96.1 % loss, and that the activity of cathepsin L decreased from 141.14 U/mL in RO to 19.64 U/mL in BO, indicating an 86.1 % reduction, due to irreversible thermal denaturation. Lastly, aspartate (Asp) and asparagine (Asn) did not exhibit significant variations across all samples, suggesting their limited contribution to umami taste of dried oysters and minimal influence from processing methods.

### 3.4 Changes in protein composition of dried oysters

To further elucidate the correlation between amino acid content variations (particularly Glu) and oyster protein degradation, the changes in protein composition across different oyster samples were analyzed as illustrated in Fig. 4B. Initially, SDS-PAGE band patterns demonstrated no significant differences between RO and BO samples, indicating that brief boiling (100 °C, 5 min) primarily induced protein denaturation (mainly conformational changes) without triggering significant substantial hydrolysis or degradation. In contrast, all protein bands in RDO-50, BDO-50, RDO-90, and BDO-90 exhibited reduced intensity, with particularly noticeable fading at 35 kDa. The band weakening was more pronounced in RDO-50 and BDO-50 compared to RDO-90 and BDO-90. These findings suggest that the drying process promoted enzymatic/thermal degradation of proteins into low-molecular-weight peptides and free amino acids (Li et al., 2022). In addition, the more intense protein degradation observed during 50 °C drying may be attributed to the activation of endogenous proteases (e.g., cathepsins and calpains) under mild drying conditions (particularly at 50 °C), which specifically cleaves peptide bonds in proteins, resulting in the disappearance of high-molecular-weight protein bands. Notably, cathepsins B/L and calpains in oysters exhibit optimal activity at 40–55 °C (Ahmed et al., 2015). During 50 °C drying of raw oysters (RDO-50), the intact enzyme system continuously hydrolyzes proteins, with the 35 kDa protein fraction undergoing preferential degradation (evidenced by extremely faint bands), due to their multiple cleavage sites. The hydrolysis products consists mainly of peptides and free amino acids, consistent with the previously observed ∼400 % surge in amino acids. Although pre-boiling of oysters inactivates most enzymes, residual enzymes (e.g., heat-resistant proteases) could still function slowly during prolonged 50 °C drying (BDO-50), resulting in band weakening comparable to that in RDO-50 (Synowiecki, 2010). The results in Fig. 4A also showed that cathepsin L still maintained a residual activity of 19.64 U/mL in BO, preserving approximately 14 % of its original function after pre-boiling. Therefore, the residual cathepsin L along with other heat-resistant proteases could slowly hydrolyze proteins during mild drying, generating small peptides and/or free amino acids.

**Fig. 5:**
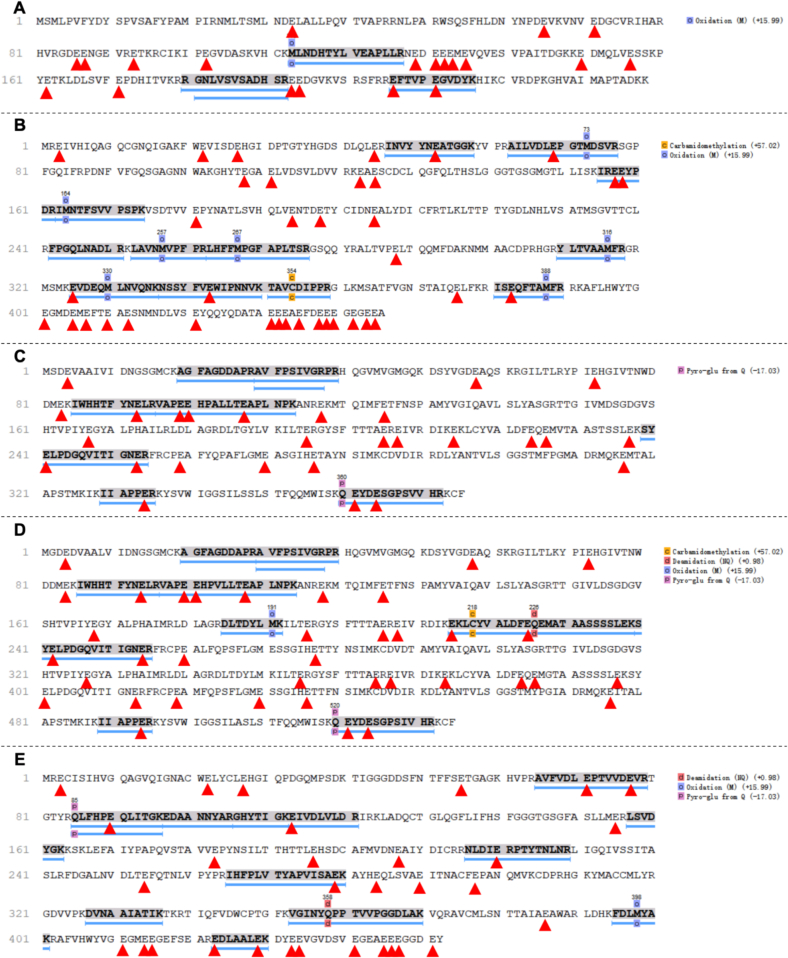
**Amino acid sequences of five proteins with high Glu contents in the 35 KDa protein band**: (A) Alpha-crystallin B chain-like, (B) Tubulin beta chain, (C) Actin cytoplasmic-like, (D) Actin-like, and (E) Tubulin alpha chain. (Note: The red triangle marking the location of Glu.) (For interpretation of the references to color in this figure legend, the reader is referred to the web version of this article.)

The 35 kDa protein band exhibited the most pronounced sensitivity to temperature variations, with particularly significant degradation observed during 50 °C drying. We therefore conducted further identification of the protein components at 35 kDa (i.e., lane 1 of RO in Fig. 4B). Mass spectrometry analysis matched a total of 55 proteins, among which 15 proteins met the high-confidence criteria (i.e., coverage ≥15 % and -10lgP ≥ 30) (Table S2). Among these 15 proteins, the top five proteins with the highest Glu content in their amino acid sequences were alpha-crystallin B chain-like (8.40 %), tubulin beta chain (8.30 %), actin cytoplasmic-like (7.20 %), actin-like (6.92 %), and tubulin alpha chain (6.86 %) (Table S2, Fig. 5).

Based on these findings, it was hypothesized that the aforementioned proteins were preferentially degraded during drying, due to their exposed hydrophilic regions containing acidic amino acids that are easily recognized by proteases. Their degradation releases substantial amounts of free Glu, making them the primary protein sources responsible for the umami taste in dried oyster products. Notably, although the standard molecular weights of *α*/*β*-tubulin (50–55 kDa) and actin-like proteins (∼42 kDa) typically exceed 35 kDa, these proteins were nevertheless identified in the 35 kDa band. Potential explanations include: a) Fresh oyster tissue contains abundant calcium-activated proteases (calpains) and cathepsins, and without protease inhibitors during sample preparation and with repeated freeze-thaw cycles, tubulin and actin may be cleaved into stable 35 kDa fragments; b) Oxidative modifications of these protein structures may reduce their SDS-binding efficiency, leading to faster electrophoretic mobility (apparent molecular weight reduction); c) These proteins might co-migrate with tropomyosin through binding interactions (Mao et al., 2018; Shi et al., 2012).

In summary, drying temperature significantly influences oyster protein degradation patterns (particularly the 35 kDa protein fraction) by regulating protease activity and protein thermal stability. The 50 °C drying process activates enzymatic cascade reactions that degrade α-crystallin, tubulin, and actin proteins, releasing Glu as the core mechanism for flavor enhancement in dried oysters. In contrast, 90 °C drying primarily induces thermal denaturation, preserving more high-molecular-weight proteins. Future research should therefore focus on assessing allergenicity of degradation fragments, screening and regulating target active proteases, precise mapping of enzymatic cleavage sites, and process optimization to achieve synergistic improvement of both flavor and safety in dried oyster products.

## 4 Conclusions

This study revealed the flavor differentiation mechanism between raw/boiled-dried oysters. Pre-boiling shifted the sour-salty profile of RO to a balanced umami-bitter base in BO, but hindered protein hydrolysis during drying, reducing Glu release by 23 % in BDO-50, cooperated to RDO-50, weakening umami. Drying temperature critically influenced flavor by modulating protein degradation. Mild drying (50 °C) in RDO-50 triggered intense protein breakdown (especially 35 kDa proteins), releasing Glu and maximizing umami, but prolonged drying increased bitterness. High-temperature drying (90 °C) reduced bitterness but diminished umami probably via protein denaturation and Maillard reactions, causing yellowing. In addition, the 35 kDa band contained Glu-rich proteins (e.g., tubulin *β*-chain, actin-like proteins), whose hydrophilic domains aided protease recognition, generating umami precursors. The degradation products of these proteins constituted the direct material basis for umami burst. In summary, for optimal flavor, raw oysters should undergo precise 50 °C drying to enhance umami. Future work should assess allergenicity of 35 kDa peptides, characterize bitter peptides, identify key protease cleavage sites, and validate variable-temperature drying for industrial-scale production.

## CRediT authorship contribution statement

**Duanquan Lin:** Writing – original draft, Visualization, Project administration, Methodology, Investigation, Funding acquisition. **Yu-Lei Chen:** Writing – review & editing, Methodology. **Wei-Sen Huo:** Methodology, Investigation. **Jing-Yi Wang:** Methodology, Investigation. **Ling-Jing Zhang:** Data curation, Conceptualization. **Jia-Yin Huang:** Writing – review & editing, Methodology. **Le-Chang Sun:** Writing – review & editing, Supervision.

## Declaration of competing interest

The authors declare that they have no known competing financial interests or personal relationships that could have appeared to influence the work reported in this paper.

## Data Availability

The data that has been used is confidential.

## Appendix A Supplementary data

Supplementary materials Image 1
