# Supplementary material for: Formation of different flavor characteristics of raw- and boiled-dried oysters
Source: Food Chem X. 2025 Jul 30;29:102854. doi: 10.1016/j.fochx.2025.102854 (PMC12355567; doi:10.1016/j.fochx.2025.102854)
Supplement: Supplementary file 1 — Supplementary materials [file mmc1.docx]

**Supplementary materials**

**Formation of different flavor characteristics of raw- and boiled-dried oysters**

Duanquan Lin^a*^, Yu-Lei Chen^a^, Wei-Sen Huo^a^, Jing-Yi Wang^a^, Ling-Jing Zhang^a^, Jia-Yin Huang^a^, Le-Chang Sun^a*^

*^a^ College of Ocean Food and Biological Engineering, Jimei University, Xiamen, China*

***Correspondence:**

Duanquan Lin

Tel: +86-15606066383

Fax: +86-592-6183955

Email: [dq. lin@jmu.edu.cn](mailto:mjcao@jmu.edu.cnAbstract)

[Le-Chang Sun](mailto:mjcao@jmu.edu.cnAbstract)

[Tel: +86-15750729546](mailto:mjcao@jmu.edu.cnAbstract)

[Email: sunlechang@jmu.edu.cn](mailto:mjcao@jmu.edu.cnAbstract)


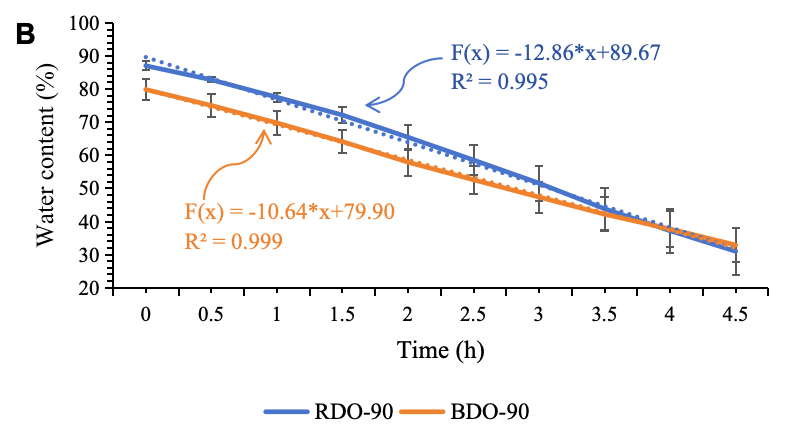

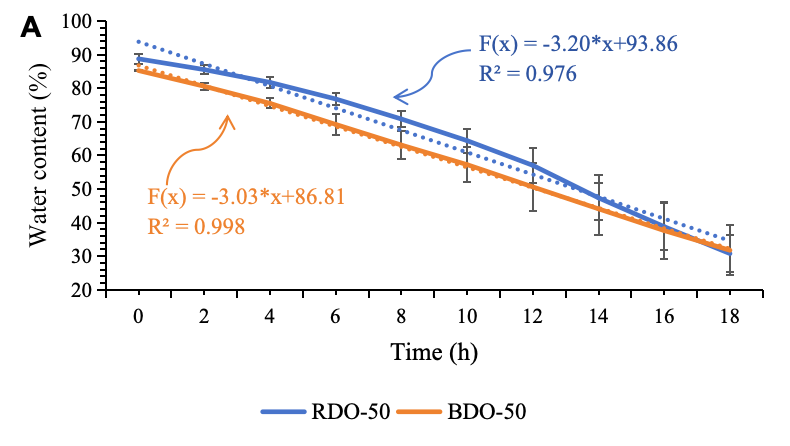


Figure S1. Drying curves of (A) raw oysters at 50°C and 90°C (i.e., RDO-50 and RDO-90) and (B) boiled oysters at 50°C and 90°C (i.e., BDO-50 and BDO-90).


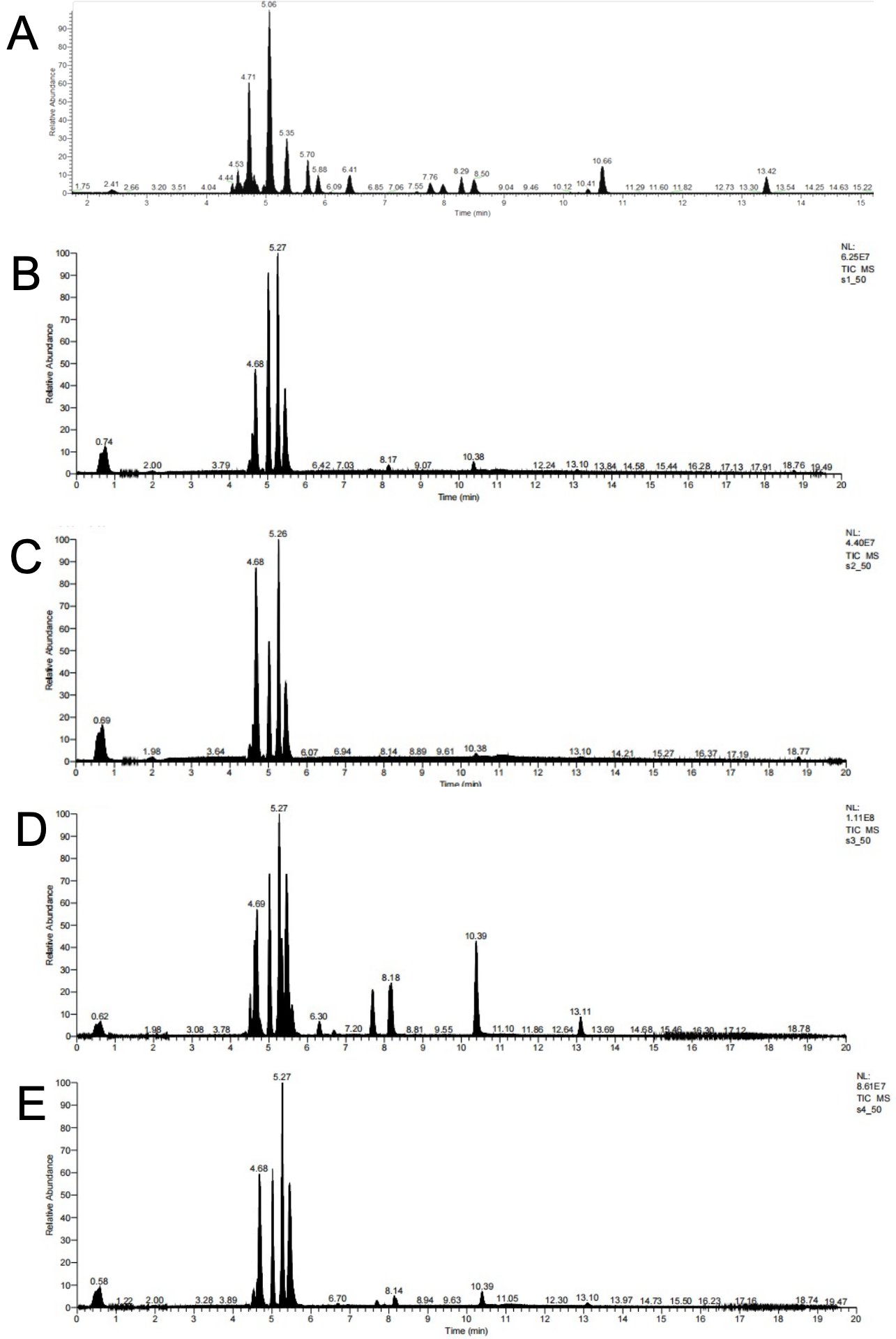


Figure S2. The chromatogram of (A) mixed standard amino acid samples, and amino acid components in oyster samples including (B) raw oyster (RO), (C) boiled oyster (BO), and their dried products under mild drying at 50°C (designated (D) RDO-50 and (E) BDO-50, respectively).

Table S1. Amino acid profiles of oyster samples including raw oyster (RO), boiled oyster (BO), and their dried products under mild drying at 50°C (designated RDO-50 and BDO-50, respectively).

Table S1. (Part A)

| Samples | Cys | Cth | PEtN | Gly | Ser | Asp | Asn | Orn | Gln | Lys | Thr | Sar | Ala |
| --- | --- | --- | --- | --- | --- | --- | --- | --- | --- | --- | --- | --- | --- |
| RO | ND | ND | 15.0 | 1470 | 8.65 | 72.6 | 15.5 | 19.4 | 40.3 | 83.8 | 36.6 | 121 | 75.80 |
| BO | ND | ND | 62.2 | 2230 | 4.58 | 97.1 | 21.0 | 25.9 | 38.2 | 132 | 16.3 | 115 | 74.70 |
| RDO-50 | 46.2 | 0.116 | 43.0 | 924 | 43.1 | 71.1 | 19.0 | 23.0 | 333 | 744 | 468 | 569 | 1190 |
| BDO-50 | ND | 0.276 | 83.7 | 1380 | 9.83 | 144 | 20.2 | 26.6 | 24.1 | 178 | 55.5 | 347 | 445.0 |

Table S1. (Part B)

| Samples | Hyp | Glu | His | 3MHis | Cit | Asa | EtN | Arg | bAla | Car | Aad | Pro | Ans |
| --- | --- | --- | --- | --- | --- | --- | --- | --- | --- | --- | --- | --- | --- |
| RO | 10.20 | 1680 | 50.80 | 1.52 | 5.60 | 23.80 | ND | 768 | 76.80 | 0.07 | 1.67 | 131.0 | ND |
| BO | 5.35 | 1980 | 41.90 | 1.46 | 8.46 | 22.30 | ND | 1490 | 81.90 | ND | 1.14 | 71.20 | ND |
| RDO-50 | 14.40 | 14100 | 248.00 | 2.65 | 6.89 | 41.00 | 0.49 | 1410 | 1200 | 0.18 | 2.26 | 679.0 | ND |
| BDO-50 | 10.60 | 11300 | 61.40 | 1.78 | 8.67 | 18.70 | 0.14 | 3120 | 486.0 | 0.05 | 2.64 | 127.0 | ND |

Table S1. (Part C)

| Samples | Hcy | Gaba | bAib | Val | Met | Hcit | Ile | Tyr | Leu | Phe | Trp | Total content |
| --- | --- | --- | --- | --- | --- | --- | --- | --- | --- | --- | --- | --- |
| RO | ND | 3.40 | 17.10 | 18.90 | 10.30 | ND | ND | 25.3 | 27.8 | 19.3 | 4.01 | 4834.22 |
| BO | ND | 3.28 | 16.40 | 16.10 | 6.22 | ND | ND | 18.7 | 15.0 | 11.6 | 2.10 | 6610.09 |
| RDO-50 | ND | 9.03 | 31.70 | 346.0 | 257 | ND | ND | 376 | 559 | 386 | 101 | 24244.12 |
| BDO-50 | ND | 7.33 | 31.70 | 39.70 | 21.40 | ND | ND | 70.3 | 77.4 | 46.9 | 32.5 | 18178.41 |

Note: 1. Sample content unit is μg/g; 2. ND indicates undetectable; 3. The original chromatograms of amino acid components were shown in Fig. S2.

Table S2. Fifteen proteins with the high-confidence criteria (i.e., coverage ≥15% and -10lgP ≥ 30) among 55 proteins identified in the 35 KDa protein band by mass spectrometry analysis.

| Protein ID | Accession | -10lgP | Coverage (%) | #Peptides | #Unique | Avg. Mass | Description | Number of total AAs | Number of Glu | Proportion (%) |
| --- | --- | --- | --- | --- | --- | --- | --- | --- | --- | --- |
| 14 | XP_052708065.1 | 236.17 | 34 | 22 | 20 | 28713 | Sperm-specific protein PHI-2B/PHI-3-like [*Magallana angulata*] | 271 | 0 | 0 |
| 8 | XP_052716215.1 | 211.95 | 50 | 13 | 13 | 36107 | Malate dehydrogenase mitochondrial-like [*Magallana angulata*] | 340 | 17 | 5.00 |
| 10 | XP_052707253.1 | 181.68 | 30 | 12 | 2 | 49936 | Tubulin beta chain [*Magallana angulata*] | 446 | 37 | 8.30 |
| 9 | XP_052681527.1 | 178.19 | 47 | 14 | 14 | 36098 | Glyceraldehyde-3-phosphate dehydrogenase-like [*Magallana angulata*] | 335 | 10 | 2.99 |
| 12 | XP_052720202.1 | 173.81 | 29 | 12 | 12 | 50161 | Tubulin alpha chain testis-specific [*Magallana angulata*] | 452 | 31 | 6.86 |
| 30 | XP_052686072.1 | 139.91 | 22 | 10 | 4 | 59332 | Actin-like [*Magallana angulata*] | 535 | 37 | 6.92 |
| 45 | \|TRYP_PIG | 138.4 | 32 | 10 | 10 | 24409 | SWISS-PROT:#CONTAM#P00761\|TRYP_PIG Trypsin - Sus scrofa (Pig). | 231 | 5 | 2.16 |
| 37 | XP_052686741.1 | 138.26 | 33 | 9 | 9 | 28710 | Proliferating cell nuclear antigen-like [*Magallana angulat*a] | 260 | 17 | 6.54 |
| 54 | XP_052712990.1 | 127.69 | 15 | 6 | 2 | 39719 | Pancreatic lipase-related protein 2-like [*Magallana angulata*] | 359 | 4 | 1.11 |
| 46 | XP_052688422.1 | 123.56 | 23 | 8 | 2 | 41657 | Actin cytoplasmic-like [*Magallana angulata*] | 375 | 27 | 7.20 |
| 57 | XP_052712993.1 | 120.65 | 16 | 5 | 1 | 40363 | Inactive pancreatic lipase-related protein 1-like isoform X2 [*Magallana angulata*] | 363 | 6 | 1.65 |
| 51 | XP_052699863.1 | 104.03 | 20 | 7 | 7 | 43118 | 60S Ribosomal protein L4-like [*Magallana angulata*] | 383 | 12 | 3.13 |
| 64 | XP_052710696.1 | 103.64 | 20 | 5 | 5 | 30969 | Proteasome subunit alpha type-1-like [*Magallana angulata*] | 280 | 18 | 6.43 |
| 119 | XP_052708874.1 | 100.05 | 16 | 4 | 4 | 27145 | Alpha-crystallin B chain-like [*Magallana angulata*] | 238 | 20 | 8.40 |
| 91 | XP_052702381.1 | 81.58 | 15 | 5 | 5 | 36688 | Retinol dehydrogenase 13-like [*Magallana angulata*] | 331 | 13 | 3.93 |
